# Three-dimensional assessment of posterior capsule–intraocular lens interaction with and without primary posterior capsulorrhexis: an intraindividual randomized trial

**DOI:** 10.1038/s41433-021-01815-4

**Published:** 2021-10-23

**Authors:** Mengting Yu, Yue Huang, Yingbin Wang, Suzhen Xiao, Xinna Wu, Wenjie Wu

**Affiliations:** Department of Ophthalmology, Provincial Clinical Medical College of Fujian Medical University, Fujian Provincial Hospital, Fuzhou, 350001 China

**Keywords:** Lens diseases, Outcomes research

## Abstract

**Purpose:**

To assess the morphologic and clinical features of posterior capsule-intraocular lens (IOL) interaction following cataract surgery with and without primary posterior continuous curvilinear capsulorrhexis (PPCCC) at a three-dimensional (3-D) level using Scheimpflug imaging.

**Methods:**

This prospective intraindividual randomized comparative study comprised 56 patients (112 eyes) with age-related cataract who had bilateral cataract surgery and hydrophobic acrylic IOLs implantation. In randomized order, cataract surgery with PPCCC was performed in 1 eye (PPCCC group), and the posterior capsule was left intact in the fellow eye (NPCCC group). Scheimpflug imaging containing 25 images distributed in 360° was taken 1 day, 1 week, 1 month, and 3 months postoperatively.

**Results:**

46 patients completed 3 months follow-up. Posterior capsule–IOL interaction can be morphologically classified into two types including complete adhesion and floppy shape in PPCCC group, and six types including full area wave, full area flat, concentric ring wave, concentric ring flat, sector, and complete adhesion in NPCCC group. The adhesion index (AI), defined as the proportion of complete adhesion of posterior capsule–IOL in 25 cross-section tomograms, was 0.45 ± 0.45, 0.79 ± 0.37, 0.92 ± 0.26 and 1.00 ± 0.00 in PPCCC group, while 0.05 ± 0.18, 0.41 ± 0.47, 0.87 ± 0.34, and 0.96 ± 0.21 in NPCCC group at 1 day, 1 week, 1 month and 3 months postoperatively, respectively (*p* = 0.001, 0.001, 0.338 and 0.151).

**Conclusions:**

3-D Scheimpflug imaging was favorable in observing of posterior capsule–IOL interaction. Faster posterior capsule adhesion to the IOL was found in PPCCC group than in NPCCC group.

## Introduction

During early phase after cataract surgery, the capsule-intraocular lens (IOL) complex experiences a significant apposition process which is crucial for IOL stability, refractive outcomes, and the development of posterior capsular opacification (PCO) [1–6]. Initially, IOL locates in a relatively large and loose capsular bag. With time, the capsular bag collapses and gradually adheres to the IOL optic [2, 4], and ultimately a firm and stable capsule–IOL complex comes into being. Within the process of capsule closure, posterior capsule–IOL adhesion plays a significant role in preventing PCO [7] and maintaining IOL stability [8]. On one hand, according to “no space, no cells, no PCO” theory, as well as previous experimental and clinical studies, a more adhesive material such as hydrophobic acrylic, could result in greater IOL optic-capsule bag adhesion [9] and less PCO formation [2, 5, 6]. On the other hand, many researchers have reported that firm contact between the capsular bag and IOL during the early postoperative period could facilitate IOL stability [8, 10]. Hence, early posterior capsule–IOL adhesion is one of the key issues in IOL in-the-bag behavior research.

Primary posterior continuous curvilinear capsulorrhexis (PPCCC) was first introduced to lower PCO [10, 11], which has been extensively adopted in congenital cataract. In these decades, growing clinical evidence has demonstrated the feasibility of PPCCC in age-related cataract and proved that PPCCC was associated with improved postoperative refractive stability [5, 12] as well as good centration of the IOL [5]. Recently, a study [13] on femtosecond laser-assisted primary posterior capsulotomy (FL-PPC) with a diameter of 3.5 mm has demonstrated no PCO occurrence throughout 6 months follow-up, while slight and incipient PCO was seen in 11 (39.28%) eyes at 6months in the control group. However, the exact mechanism of less PCO formation and better IOL stability in eyes with posterior capsulotomy remained unknown. Perhaps in these eyes with posterior capsular opening, the reduced area of posterior capsule might proceed the posterior capsule–IOL adhesion, which might facilitate PCO prevention and IOL stability. To our knowledge, no studies have investigated the process of capsule–IOL interaction and adhesion in eyes with PPCCC, which might be critical to understand the underlying mechanisms.

Numerous methods have been adopted to explore how the posterior capsule–IOL interaction evolved over time and demonstrated that posterior capsule–IOL adhesion was closely related to the development of PCO and IOL stability [1, 14, 15]. However, most previous methods investigated the evolution of capsule–IOL complex configurations only at horizontal or vertical meridian and limited in moderately dilated pupils [1, 3, 15]. Recently, the introduction of the latest generation of Pentacam AXL (Oculus, Wetzlar), which is equipped with a rotating Scheimpflug camera, allows rapid acquisition of three-dimensional (3-D) IOL–capsule complex configurations with more comprehensive and detailed information in even moderately dilated pupils.

Hence, using Scheimpflug imaging methods, we conducted a prospective intraindividual randomized comparative clinical trial to further explore the morphologic evolution process and clinical features of posterior capsule–IOL interaction in eyes with and without PPCCC after cataract surgery at 3-D level.

## Methods

### Participants

This prospective intraindividual double-blinded randomized comparative clinical study was conducted at the Department of Ophthalmology, Fujian Provincial Hospital, Fuzhou, China, from May 2020 to February 2021 and in accordance with the tenets of the Declaration of Helsinki. This study enrolled patients who had bilateral cataract surgery with implantation of one-piece 360° square-edged hydrophobic IOLs with a length of 13 mm (Tecnis ZCB00, Abbott Medical Optics or Proming A1-UV, Eyebright). In randomized order, cataract surgery with PPCCC was performed in one eye, and the posterior capsule was left intact in the fellow eye. All IOLs were implanted in the capsular bag in both groups.

The inclusion criteria were: (1) a diagnosis of bilateral age-related cataract; (2) corneal astigmatism of less than 1.50 D; (3) the axial length between 22 mm and 26 mm;(4) being scheduled for second-eye surgery within 1 month after the first-eye surgery. Patients with corneal pathology, uveitis, glaucoma, pseudoexfoliation, strabismus, history of ocular trauma, and retinal pathology were excluded. The study protocol was approved by the Institutional Review Board of Fujian Provincial Hospital. All participants granted us informed written consent prior to cataract surgery. The study was registered at Chinese Clinical Trial Register Center (ChiCTR-2000033304).

### Randomization

In all cases, the second eye operation was performed within 1 month after the first operation. Randomization will be determined with opaque sealed envelopes containing a card labeled “PPCCC” or “NPCCC.” The data analyzer randomly picked and opened one of two envelopes at the patient’s last visit prior to the first eye operation. The surgeon was masked to group allocation until the time before surgery, while the patients and the examiners were masked to randomization all the time.

### Surgical technique

All surgeries were performed by one experienced surgeon (WJ. W) using a standard phacoemulsification technique. A 2.4-mm clear cornea incision was made, followed by the continuous curvilinear capsulorhexis with a diameter of 5.5 mm, nucleus removal, cortical aspiration, and posterior capsular polishing. The following procedures were dependent on the groups. In PPCCC group, the posterior capsule was punctured with a 22-gauge needle in the center of the posterior capsule and a fissure about 1 mm length was created after being filled with ophthalmic viscosurgical device (OVD) (sodium hyaluronate 15 mg/ml, Qisheng Biological Preparation Co., Ltd). Next, OVD was rejected into the capsular bag again. Then, PPCCC was conducted using capsular forceps and a well-centered round posterior opening with approximate diameter of 4.0 mm was created. After refilling the capsular bag with OVD, the IOL was then inserted into the bag. The residual OVD was aspirated and the surgical wounds were watertight.

In NPCCC group, after cortex removal and capsular polish, the IOL was inserted into the capsular bag. Patients with iatrogenic posterior capsule rupture or tear and obvious posterior capsular plaque during surgery were not included.

All patients attended scheduled control visits at 1 day, 1 week, 1 month, and 3 months postoperatively. Each examination included visual acuity, objective refraction, and dilated Scheimpflug imaging.

### Visual acuity and refractive error

Total refractive error was measured with an auto refractometer (Auto Ref/Keratometer ARK-1a, NIDEK). The spherical equivalent (SE) value was determined as the sum of the spherical power with half of the cylindrical power. The refractive prediction error (RPE) was calculated by subtracting the estimated preoperative SE from the postoperative SE. Corrected distance visual acuity (CDVA) was obtained using a Snellen chart and converted to logarithm minimal angle resolution for statistical analysis.

### Postoperative measurements by Scheimpflug system

After full mydriasis using a mixture of 0.5% phenylephrine and 0.5% tropicamide (Mydrin-P, Santen), Pentacam examination was performed under the standard dim-light conditions. The data collected by the Scheimpflug system were adopted only when the data quality statement was “OK”. In each acquisition, the rotating Scheimpflug camera captured 25 images distributed in 360° automatically. In the mode of 3-D Scheimpflug Image Overview, 25 images were overviewed to assess the overall morphologic characteristics of the posterior capsule–IOL interaction. Besides, additional cross-sectional images of the anterior chamber and the capsule–IOL complex were obtained at the horizontal meridian for anterior chamber depth (ACD) analysis. All the measurements were taken at least twice consecutively.

The postoperative ACD, defined as the distance between the posterior corneal surface and anterior IOL surface, was manually measured after adjusting the contrast of the Scheimpflug image at horizontal meridian in both groups.

### Statistical analyses

The data were presented as the mean ± standard deviation. The significance of between-group differences was determined using the paired *t* test if the data were normally and equally distributed. If not, the Manne-Whitney rank-sum test was used. Categorical variables were presented as counts and percentages and compared with the Chi-square test when appropriate (expected frequency > 5). Otherwise, the Fisher exact test was used. Repeated-measures analysis of variance was performed to compare clinical conditions within the same subjects at different time points. All statistical analyses were performed using SPSS (version 24, SPSS, Inc.). A *P* value < 0.05 was considered statistically significant.

## Results

### Demographic data

During the study, 56 patients were included, and 10 patients did not attend complete scheduled follow-up. Therefore, 46 patients completed three months follow-up, which was available for analysis. At enrollment, there were no significant ocular differences between NPCCC eyes and PPCCC eyes (Table 1; *P* > 0.05). No serious postoperative complications, such as vitreous prolapse or subsequent retinal morbidity, were observed in subjective slit-lamp examination and no patient developed severe PCO in need of neodymium–yttrium aluminum garnet laser capsulotomy during the 3 months follow-up period in either group.

**Table 1:** Baseline demographic and ocular characteristics of enrolled patients^a^.

	PPCCC group	NPCCC group	*P* value
Mean age (years)	69.13 ± 6.05		
Gender (male/female)	13/33		
IOL (ZCB00/ A1-UV)	39/7		
Eye (right/left)	27/19		
AXL (mm)	23.36 ± 0.81	23.32 ± 0.82	0.514
CDVA (logMAR)	0.82 ± 0.62	0.85 ± 0.55	0.724
IOP (mmHg)	15.98 ± 2.11	15.99 ± 2.41	0.968
ACD (mm)	3.03 ± 0.32	3.07 ± 0.36	0.098
Km (D)	44.25 ± 1.32	44.21 ± 1.31	0.629
Cylinder(D)	−0.75 ± 0.53	−0.78 ± 0.33	0.766
Target refraction(D)	−0.38 ± 0.22	−0.35 ± 0.28	0.464

*PPCCC* primary posterior continuous curvilinear capsulorrhexis, *NPCCC* without primary posterior continuous curvilinear capsulorrhexis, *AXL* axial length, *CDVA* corrected distance visual acuity, *IOP* intraocular pressure, *ACD* anterior chamber depth, *Km* mean corneal curvature.

^a^Paired *t* test.

### Visual acuity, spherical equivalent, and refractive prediction error

Three months after surgery, the CDVA significantly improved from 0.85 ± 0.55 (range from 0.39 to 3.0) preoperatively to 0.03 ± 0.07 (range from −0.08 to 0.30) (*P* < 0.001) in NPCCC group, and 0.82 ± 0.62 (range from 0.30 to 3.0) to 0.04 ± 0.09 (range from −0.08 to 0.30) (*P* < 0.001) in PPCCC group. There was no difference between two groups 3 months after surgery. There was no significant difference in CDVA, SE, and RPE between the two groups at any time point (Table 2).

**Table 2:** Visual acuity, refractive error, anterior chamber depth, adhesion index, and percentage of eyes with complete posterior capsule–intraocular adhesion in two groups over time.

	PPCCC group	NPCCC group	*P* value
CDVA (logMAR)			
1 day	0.06 ± 0.10	0.06 ± 0.10	0.339^a^
1 week	0.05 ± 0.10	0.03 ± 0.09	0.122^a^
1 month	0.05 ± 0.10	0.04 ± 0.09	0.690^a^
3 months	0.04 ± 0.09	0.03 ± 0.07	0.158^a^
SE (D)			
1 day	−0.37 ± 0.08	−0.25 ± 0.09	0.109^a^
1 week	−0.42 ± 0.07	−0.39 ± 0.09	0.647^a^
1 month	−0.45 ± 0.09	−0.32 ± 0.08	0.198^a^
3 months	−0.34 ± 0.09	−0.32 ± 0.08	0.831^a^
RPE (D)			
1 day	−0.01 ± 0.53	0.08 ± 0.60	0.231^a^
1 week	−0.08 ± 0.47	−0.05 ± 0.54	0.634^a^
1 month	−0.14 ± 0.65	−0.00 ± 0.47	0.093^a^
3 months	0.05 ± 0.56	0.03 ± 0.50	0.277^a^
ACD (mm)			
1 day	4.09 ± 0.31	4.16 ± 0.32	0.003^a^
1 week	3.99 ± 0.33	4.03 ± 0.31	0.010^a^
1 month	4.01 ± 0.32	4.01 ± 0.31	1.000^a^
3 months	4.03 ± 0.32	3.99 ± 0.30	0.019^a^
AI			
1 day	0.45 ± 0.45	0.05 ± 0.18	0.001^a^
1 week	0.79 ± 0.37	0.41 ± 0.47	0.001^a^
1 month	0.92 ± 0.26	0.87 ± 0.34	0.338^a^
3 months	1.00 ± 0.00	0.96 ± 0.21	0.151^a^
Percentage of CA			
1 day	13 (28.26%)	1 (2.17%)	0.001^b^
1 week	33 (71.74%)	16 (34.78%)	0.001^c^
1 month	41 (89.13%)	39 (84.78%)	0.758^c^
3 months	46 (100%)	44 (95.65%)	0.495^b^

Data are expressed as the mean ± SD.

*PPCCC* primary posterior continuous curvilinear capsulorrhexis, *CDVA* corrected visual acuity, *SE* spherical equivalent, *D* diopter, *RPE* refractive prediction error, *ACD* the distance between the posterior corneal surface and anterior IOL surface, *AI* adhesion index, *CA* complete adhesion.

^a^Repeated-measures analysis.

^b^Fisher exact test.

^c^Chi-square test.

### Axial shift of the intraocular lens

The preoperative ACD measurements were statistically comparable between PPCCC eyes and NPCCC eyes (*P* = 0.098). Compared to 1 day postoperatively, ACD decreased at the first week after surgery (*P* < 0.001) in both groups, and then remained stable for 3 months (Table 2 and Figure S1 in the Supplementary Material).

### Morphologic changes in posterior capsule–IOL interaction over time

**Fig. 1:**
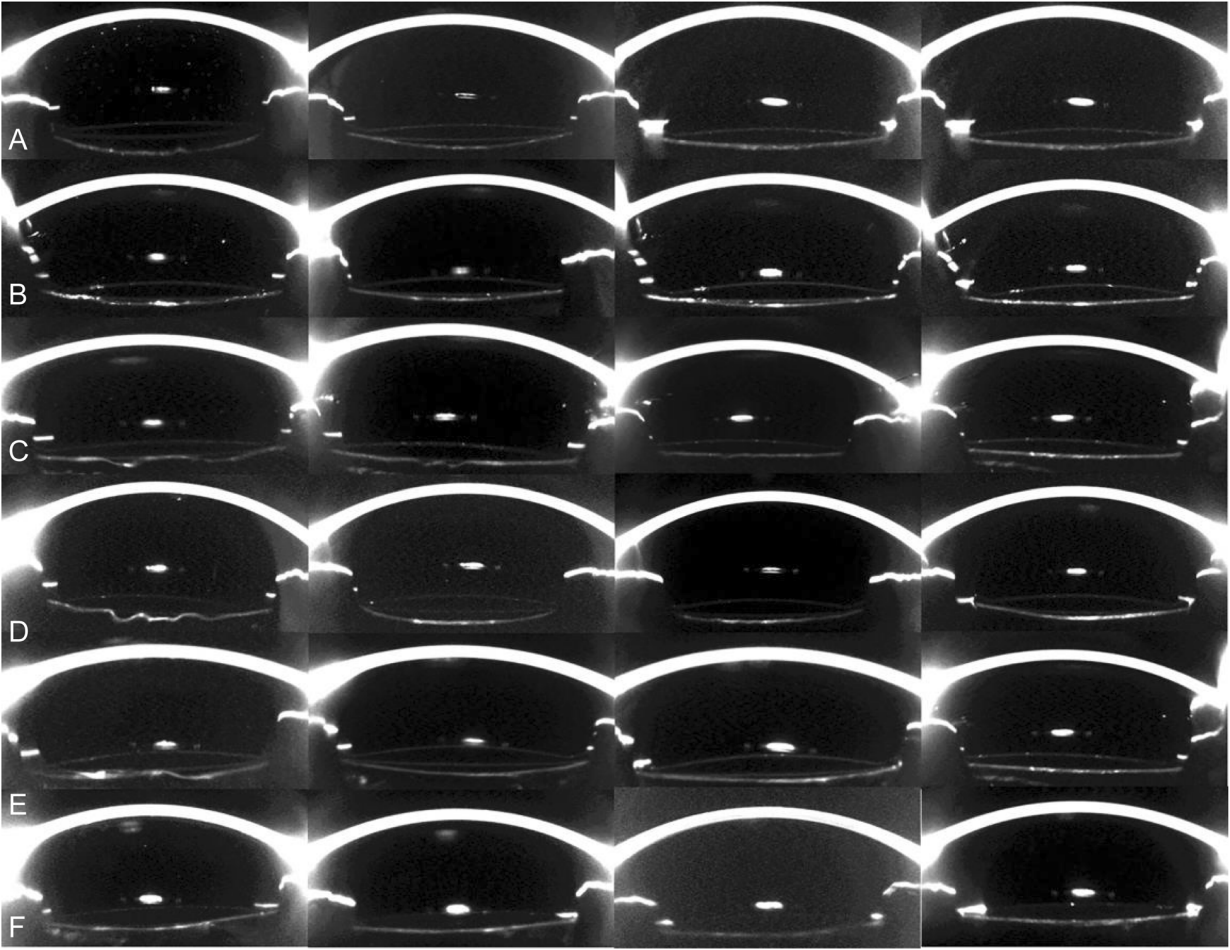
Morphologic changes in posterior capsule–IOL interaction over time in eyes with and without posterior continuous curvilinear capsulorrhexis. **a** Full and flat capsule–IOL inadhesion gradually changed into complete adhesion. **b** Capsule–IOL inadhesion was observed only in the center aera of the capsule one day postoperatively. With time, this concentric and flat inadhesion gradually changed into complete adhesion. **c** Full and wave capsule inadhesion changed into complete adhesion. **d** Concentric ring wave capsule shape changed to full area flat shape, and finally concentric ring flat inadhesion occurred. **e** Sector capsule–IOL inadhesion changed to full and flat shape, and finally, completely adhesion formed. **f** Capsule–IOL completely adhesion from the beginning.

In NPCCC group, six major types of posterior capsule–optic interaction were observed on the first day after surgery, according to the morphologic classification system described in Zhu’s previous study [16]: full area wave (4/46, 8.70%), full area flat (2/46, 4.35%), concentric ring wave (25/46, 54.35%), concentric ring flat (8/46,17.40%), sector (3/46,6.52%) and complete adhesion (4/46, 8.70%) (Fig. 1A–F). Generally, more wave-shaped (63.05%) than flat-shaped (21.75%) configurations were presented in NPCCC group. With the elapse of time, the space between IOL and posterior capsule decreased and finally disappeared in most eyes. Wave-shaped configurations in seven patients (15.21%) were observed to transform to flat-shaped initially and then completed adhesion. The morphologic changes in posterior capsule–IOL interaction over time are summarized in Table S1 in the Supplementary Material.

**Fig. 2:**
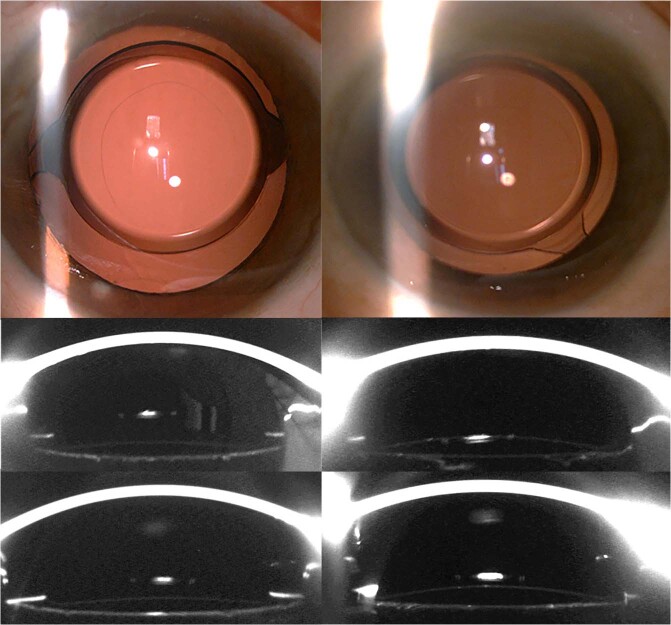
Morphologic changes in posterior capsule–intraocular lens (IOL) interaction over time in eyes with posterior continuous curvilinear capsulorrhexis (PPCCC). Two types including complete adhesion (left) and floppy shape (right) in PPCCC group. (Top row, left and right) In eyes with posterior continuous curvilinear capsulorrhexis, a round opening was observed a day after the surgery. The complete adhesion of IOL and posterior capsule was observed 1 day (Middle row, left) and 1 week (Bottom row, left) after surgery, with the remaining posterior capsule edge closely attached to the IOL. The floppy shape posterior capsule was observed 1 day after surgery (Middle row, right) and the remaining posterior capsule gradually attached to the IOL 1 week after surgery (Bottom row, right).

In PPCCC group, two major capsule–IOL configurations were observed on the first day after surgery (Fig. 2): complete adhesion (78.26%) and floppy shape (21.74%). Floppy shape was defined as posterior capsulorhexis margin in contact with the IOL optic. Most PCCC rim gradually came into contact with the optic with time, and complete adhesion was observed in 41 (89.13%), 43 (93.48%), 46 (100%) eyes on 1 week, 1 month, 3 months, postoperatively.

### Adhesion index (AI) of the posterior capsular–IOL

**Fig. 3:**
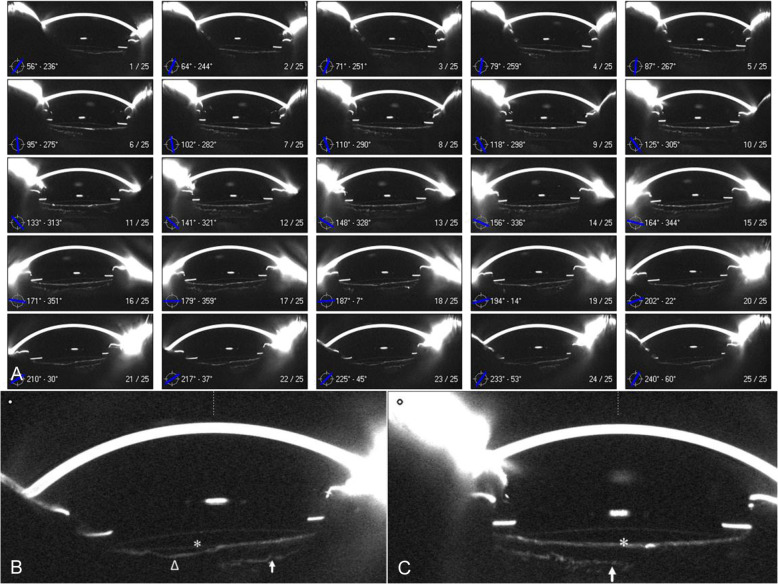
Scheimpflug image overview of a representative patient at postoperative 1 week. **a** Scheimpflug image overview contained 25 cross-sectional tomographs at 25 dimensions, where incomplete adhesion was observed in 15 dimensions, while complete adhesion was observed in ten dimensions, hence the AI value was 10/25 = 0.4. **b** Complete adhesion was observed at 125°. **c** Sector posterior shape was observed at 225°. Posterior capsule (white triangle), posterior surface of IOL (white asterisk) and anterior hyaloid membrane (white arrow).

Complete adhesion (CA), known as the complete adhesion of the capsule to posterior surface of the IOL optic. In the mode of 3-D Scheimpflug image overview containing 25 dimensions, the adhesion index (AI) was defined as the following formula (Fig. 3A):

$${{{{{{{\mathrm{AI}}}}}}}} = {{{{{{{\mathrm{NCA}}}}}}}}/25$$

Where NCA is the number of complete adhesions in 25 images.

In NPCCC group, AI was 0.05 ± 0.18, 0.41 ± 0.47, 0.87 ± 0.34 and 0.96 ± 0.21, while in PPCCC group, AI was 0.45 ± 0.45, 0.79 ± 0.37, 0.92 ± 0.26 and 1.00 ± 0.00 at 1 day, 1 week, 1 month and 3 months after surgery, respectively (*p* = 0.001, 0.001, 0.338 and 0.151, Table 2).

Complete Posterior capsule–IOL adhesion (AI = 1) was observed in 13(28.26%), 33(71.74%), 41(89.13%) and 46(100%) eyes 1 day, 1 week, 1 month and 3 months postoperatively in PPCCC group, while the proportion was 1(2.17%), 16(34.78%), 39(84.78%) and 44(95.65%) in NPCCC group, respectively, which was much lower than PPCCC group (*P* = 0.028, 0.005, 1.000 and 0.240).

## Discussion

Many researchers have reported that firm contact between the capsular bag and IOL during the early postoperative period could inhibit the migration of LECs [1–3, 7] and facilitate IOL stability [8]. Currently, evidence of PPCCC related to improved postoperative refractive stability, good centration of the IOL, and prevention of PCO is accumulating [5, 12, 17, 18], which may have potential benefits in premium IOLs including multifocal, toric, and extended depth of focus ones. However, the IOL–capsule complex evolution in PPCCC eyes has not been fully elucidated, which appeared to be significant for understanding the underlying mechanisms of better IOL stability and lower PCO incidence. To the best of our knowledge, this was first prospective, randomized, and controlled fellow eye study to investigate the posterior capsule–IOL interaction in eyes with and without PPCCC in the early postoperative phase over 1 day, 1 week, 1 month, and 3 months postoperatively.

Previous investigators had cast their interests and explorations on the early capsule–IOL complex apposition process after conventional cataract surgery with IOL implantation [1–4, 6, 16]. Sacu et al. [3] assessed capsule–IOL apposition with time-domain optical coherence tomography (OCT), whose inherent limitation was that most anterior segment OCTs were designed to image the cornea only and could not provide an entire anterior segment view including cornea, iris, and IOL at one scan. Zhu et al. [16] evaluated posterior capsule-optic adhesion of different intraocular lenses using Scheimpflug imaging only at horizontal scan. These previous researches of capsular dynamics were usually conducted at the level of 2-D, which were limited to a single cross-section tomogram, and thus might miss some information of other dimensions in the evolution of capsule–IOL complex. In these years, Yu et al. [2] adopted the advanced generation OCT (high-speed swept-source OCT) to investigate the posterior capsular behavior at 3-D level. However, it was a retrospective study and evaluated only a single timepoint at 2 years postoperatively, which limited its validity. In this study, we utilized Scheimpflug method to image the posterior capsule–IOL interaction, which permitted whole anterior segment imaging at one scan, and provided 25 cross-sectional images distributed in 360°. At 25 different dimensions, we found posterior capsule–IOL adhesion did not proceed at the same speed (Fig. 3), which suggested routine single cross-sectional image was insufficient to provide comprehensive details of the time-dependent changes. Thus, according to 3-D Scheimpflug imaging, we characterized the posterior capsule–IOL adhesion in 25 images by adhesion index (AI) and further compared the differences of AI in PPCCC group and NPCCC group at scheduled visits to quantify the extent of posterior capsule–IOL interaction. A higher AI value meant more posterior capsule–IOL adhesion at 25 dimensions.

In NPCCC group, 1 week after the surgery, only 16 (34.78%) eyes achieved complete adhesion in 25 cross-sectional tomographs (AI = 1) and the mean AI was 0.41 ± 0.47. However, Sacu [3] and his associates observed complete adhesion of posterior capsule to the acrylic IOL optic surface at horizontal meridian within approximately 7 days using time-domain OCT. This discrepancy might result from two aspects. On one hand, high resolution of Scheimpflug imaging used in our study might better reveal those subtle space between the posterior capsule and IOL optic than the time-domain OCT used in Sacu’s study. Zhu et al. [16] also reported posterior capsule–IOL inadhsion was observed in 33% patients with acrylic hydrophilic IOL at the same time point using high-resolution Scheimpflug imaging. On the other hand, the mean AI (0.41 ± 0.47) from 25 cross-sectional images contained more information on capsule–IOL interaction of comprehensive directions than at a single meridian. Furthermore, the no-space, no-cells concept [19] implies that early contact between the posterior capsule and IOL optic might result in a lower incidence of PCO. Liu et al. [20] reported that in their in vitro model, a confluent monolayer of LECs over the posterior capsule was seen after 7.2 ± 0.7 days in patients older than 60 years. Thus, timely complete capsule–IOL adhesion within one week might play an important role in the prevention of PCO. In our study, on the postoperative 1 day and 1 week, 8 (21.62%) and 24 (64.86%) eyes achieved complete adhesion (AI = 1) in PPCCC group while 1(2.7%) and 12(32.43%) eyes in NPCCC group (*P* = 0.028 and *P* = 0.005). Meanwhile, AI was higher in PPCCC group than that in NPCCC group (*P* = 0.001 and 0.001). Consequently, we speculated that a higher AI in PPCCC group represented earlier and larger scale adhesion of posterior capsule–IOL, which might facilitate the prevention of LECs migration within 1 week. Thereafter, the opacity of the remaining posterior capsule posterior to the IOL optic in PPCCC eyes might also be reduced, which was supported by Schojai’s study [13], who reported no PCO occurrence in eyes with FL-PPC throughout 6 months follow-up, while PCO was seen in 11 (39.28%) eyes in the control group. Therefore, we would continue our follow-up observation on PCO to further identify the difference of peripheral posterior capsule opacification in two groups.

Three months after surgery, complete capsule–IOL adhesion in 25 cross-sectional images was observed in vast majority eyes (44/46, 95.66%) in NPCCC group in our study. However, minor capsule–IOL inadhesion still presented in two diabetic NPCCC eyes (Fig. 1D) in all 25 images 3 months after surgery, while the fellow PPCCC eyes achieved complete adhesion at 1 day after surgery, which could also be seen in 13(28.26%) eyes in PPCCC group. Therefore, we postulated that in diabetic patients who were more prone to PCO and with larger capsular bags, performing PPCCC might be of more significance compared to conventional procedures that preserved intact capsule bag. The exact reason of the long-term inadhesion in these two diabetic NPCCC eyes remained unclear. Long-term follow-up with larger sample size of diabetic eyes is required to determine whether the minor gap would exist persistently and the occurrence of PCO.

The morphologic features of posterior capsule–IOL configurations in two groups were variable. In PPCCC group, two main types of configurations were seen: complete adhesion and floppy shaped (Fig. 2). The former one was more common (71.74%) than the latter one (28.26%) at postoperative day 1. With time, the remaining posterior capsule gradually attached to the posterior optic surface in all eyes at 3 months after surgery. In NPCCC group, we categorized the configurations of posterior capsule into six major types based on the classification system described in Zhu’s previous study [16]: full area wave, full area flat, concentric ring wave, concentric ring flat, sector, and complete adhesion (Fig. 1). On postoperative day 1, the proportions of different morphology were as follows: concentric ring wave in 25 (54.35%) eyes, concentric ring flat in 8 (17.40%) eyes, complete adhesion in 4 (8.70%) eyes, sector in 3 (6.52%) eyes, full area wave in 4 (8.70%) eyes, full area flat in 2 (4.35%) eyes. Generally, wave shape (63.04%) was more common than flat shape (21.74%), which was in agreement with Zhu’s study [16], who mainly focused on the posterior capsule adhesion to IOLs with different materials and designs at a single meridian. This wave morphologic feature indicated that the capsule bag was relatively large for IOLs in most eyes 1 day postoperatively. At the last follow-up, flat concentric inadhesion was observed in only two eyes in NPCCC group, whereas in the rest of eyes, posterior capsule approached the rear surface of IOL optic and then matched the latter’s shape, and finally, the completed adhesion formed. An interesting phenomenon was observed in 6 eyes (16.21%) that wave-shaped posterior capsule transformed into flat-shaped (Fig. 1D) initially and then into complete adhesion. The morphological changes over time in PPCCC eyes and NPCCC eyes may be associated with relatively large capsular bag shrinkage and reshape, and ultimately, a stable IOL–capsule complex formed in 46 (100%) eyes in PPCCC eyes and 44 (95.65%) in NPCCC eyes 3 months postoperatively. Faster posterior capsule–IOL adhesion was observed in PPCCC group than NPCCC group, which probably suggested that earlier adhesion and formation of firm capsule–IOL complex in PPCCC group lead to better IOL stability. The feature might be of significant value in IOLs that required stable in-the-bag position especially for toric IOLs and regional refractive IOLs.

The precise postoperative refractive outcome was greatly dependent on the position of the IOL in the eye, which was usually predicted by the ACD [5, 12]. Changes in ACD might lead to unexpected refractive surprise, which was mainly presumed to be caused by capsule shrinkage and fibrosis. The centripetal force toward the center of the CCC is converted to anteriorly directed forces on IOL by the taut posterior capsule. Previous studies showed that compared to 1 day postoperatively, the ACD became shallower overtime after cataract surgery, and remained stable 3 month postoperatively after conventional cataract surgery [6]. In our study, we also observed a similar tendency in NPCCC group. Compared to NPCCC eyes, less IOL axial anterior movement within 3 months was found in PPCCC eyes though without significance (Figure S1 in the Supplementary Material and Table 2). A flatter ACD curve was observed in PPCCC group, in accordance with Kim’s study, which might indicate early capsule–IOL adhesion promote IOL axial stability.

Strengths of this study included the prospective, double-blinded randomized controlled individual study design, and that the study was designed, conducted, and analyzed according to a pre-specified protocol. However, there are several potential limitations of this study. First, we evaluated the process for 3 months after surgery in both groups and observed better adhesion in PPCCC eyes during early postoperative period. However, longer-term follow-up is still needed to observe its clinical relevance in terms of long-term refractive outcomes, IOL stability as well as PCO. Furthermore, we are observing the patients for further follow-up to clarify the relationship of the speed of capsule–IOL adhesion and PCO in both groups. Second, this study involved a limited number of patients. We are recruiting more patients for further follow-up. Although we did not focus on the process of capsular bend and anterior capsule apposition to IOLs which had been studied in abundant literatures, we assessed the morphologic and clinical features of posterior capsule–IOL interaction at 3-D level, which was a complement to the previous researches on posterior capsule–IOL interaction and the database of capsule bag-IOL apposition behavior.

In conclusion, 3-D Scheimpflug imaging was favorable in the observation of posterior capsule–IOL interaction in eyes with and without PPCCC. The technique captured both cross-sectional tomograms and 25 images distributed at 360° at one scan. We found faster posterior capsule adhesion to the IOL in PPCCC than in NPCCC group. Besides, we observed various morphologic types of posterior capsule–IOL interaction in both groups over time. Longer follow-up observation and future studies on the relationship between PCO and posterior capsule–IOL adhesion in PPCCC and NPCCC eyes may be necessary.

### Summary

#### What was known before

- Evidence of primary posterior continuous curvilinear capsulorrhexis (PPCCC) related to improved postoperative refractive stability, good centration of the IOL, and prevention of PCO is accumulating, but the exact mechanisms remained unknown.

#### What this study adds

- For the first time, using Scheimpflug imaging, capsule–IOL complex interaction at a three-dimensional level was observed in eyes with and without PPCCC over 1 day, 1 week, 1 month, and 3 months postoperatively, and faster posterior capsule adhesion to the IOL was observed in eyes with PPCCC than in the control group.

## Supplementary information

Morphologic changes in posterior capsule-optic interaction over time in NPCCC group

Changes in postoperative anterior chamber depth in eyes with and without posterior continuous curvilinear capsulorrhexis over time

## References

[1] Zhao Y, Li J, Lu W, Chang P, Lu P, Yu F (2013). Capsular adhesion to intraocular lens in highly myopic eyes evaluated in vivo using ultralong-scan-depth optical coherence tomography. Am J Ophthalmol.

[2] Fang Y, Xixia D, Jin L, Lei L, Pingjun C, Hongfang Z (2020). Relationship of posterior capsular opacification and capsular bend type investigation based on swept-source optical coherence tomography. Curr Eye Res.

[3] Sacu S, Findl O, Linnola RJ (2005). Optical coherence tomography assessment of capsule closure after cataract surgery. J Cataract Refract Surg.

[4] Hayashi H, Hayashi K, Nakao F, Hayashi F (2002). Elapsed time for capsular apposition to intraocular lens after cataract surgery. Ophthalmology.

[5] Kim KH, Kim WS (2010). Intraocular lens stability and refractive outcomes after cataract surgery using primary posterior continuous curvilinear capsulorrhexis. Ophthalmology.

[6] Ding X, Wang Q, Xiang L, Chang P, Huang S, Zhao YE (2020). Three-dimensional assessments of intraocular lens stability with high-speed swept-source optical coherence tomography. J Refract Surg.

[7] Oshika T, Nagata T, Ishii Y (1998). Adhesion of lens capsule to intraocular lenses of polymethylmethacrylate, silicone, and acrylic foldable materials: an experimental study. Br J Ophthalmol.

[8] Nagata T, Minakata A, Watanabe I (1998). Adhesiveness of AcrySof to a collagen film. J Cataract Refract Surg.

[9] Lombardo M, Carbone G, Lombardo G, De Santo MP, Barberi R (2009). Analysis of intraocular lens surface adhesiveness by atomic force microscopy. J Cataract Refract Surg.

[10] Linnola RJ, Sund M, Ylönen R, Pihlajaniemi T (2003). Adhesion of soluble fibronectin, vitronectin, and collagen type IV to intraocular lens materials. J Cataract Refract Surg.

[11] Linnola RJ, Werner L, Pandey SK, Escobar-Gomez M, Znoiko SL, Apple DJ (2000). Adhesion of fibronectin, vitronectin, laminin, and collagen type IV to intraocular lens materials in pseudophakic human autopsy eyes. Part 2: explanted intraocular lenses. J Cataract Refract Surg.

[12] Stifter E, Menapace R, Luksch A, Neumayer T, Sacu S (2008). Anterior chamber depth and change in axial intraocular lens position after cataract surgery with primary posterior capsulorhexis and posterior optic buttonholing. J Cataract Refract Surg.

[13] Schojai M, Schultz T, Haeussler-Sinangin Y, Boecker J, Dick HB (2017). Safety of femtosecond laser-assisted primary posterior capsulotomy immediately after cataract surgery. J Cataract Refract Surg.

[14] Haripriya A, Chang DF, Vijayakumar B, Niraj A, Shekhar M, Tanpreet S (2017). Long-term posterior capsule opacification reduction with square-edge polymethylmethacrylate intraocular lens: randomized controlled study. Ophthalmology.

[15] Cheng JW, Wei RL, Cai JP, Xi GL, Zhu H, Li Y (2007). Efficacy of different intraocular lens materials and optic edge designs in preventing posterior capsular opacification: a meta-analysis. Am J Ophthalmol.

[16] Zhu X, He W, Yang J, Hooi M, Dai J, Lu Y (2016). Adhesion of the posterior capsule to different intraocular lenses following cataract surgery. Acta Ophthalmol.

[17] Al-Nashar HY, Khalil AS (2016). Primary posterior capsulotomy in adults with posterior capsule opacification. J Cataract Refract Surg.

[18] Galand A, van Cauwenberge F, Moosavi J (1996). Posterior capsulorhexis in adult eyes with intact and clear capsules. J Cataract Refract Surg.

[19] Apple DJ, Solomon KD, Tetz MR, Assia EI, Holland EY, Legler UF (1992). Posterior capsule opacification. Surv Ophthalmol.

[20] Liu CS, Wormstone IM, Duncan G, Marcantonio JM, Webb SF, Davies PD (1996). A study of human lens cell growth in vitro. A model for posterior capsule opacification. Investig Ophthalmol Vis Sci.

