# Supplementary material for: Three-dimensional assessment of posterior capsule–intraocular lens interaction with and without primary posterior capsulorrhexis: an intraindividual randomized trial
Source: Eye (Lond). 2021 Oct 23;36(11):2130–6. doi: 10.1038/s41433-021-01815-4 (PMC9581986; doi:10.1038/s41433-021-01815-4)
Supplement: Supplementary file 1 — Morphologic changes in posterior capsule-optic interaction over time in NPCCC group [file 41433_2021_1815_MOESM1_ESM.docx]

**Table S1** Morphologic changes in posterior capsule-optic interaction over time in NPCCC group

|  | 1 day | 1 week | 1 month | 3 months |
| --- | --- | --- | --- | --- |
| full area wave | 4 (8.70%) | 0 (0%) | 0(0%) | 0(0%) |
| full area flat | 2 (4.35%) | 3 (6.52%) | 1(2.17%) | 0(0%) |
| concentric ring wave | 25 (54.35%) | 9 (19.57%) | 2(4.34%) | 0(0%) |
| concentric ring flat | 8 (17.40%) | 10 (21.74%) | 4(8.70%) | 2(4.34%) |
| sector | 3 (6.52%) | 0(0%) | 0(0%) | 0(0%) |
| complete adhesion | 4(8.70%) | 24(52.17%) | 39(84.78%) | 44(95.65%) |

NPCCC= without posterior continuous curvilinear capsulorrhexis
