# Supplementary material for: Three-dimensional assessment of posterior capsule–intraocular lens interaction with and without primary posterior capsulorrhexis: an intraindividual randomized trial
Source: Eye (Lond). 2021 Oct 23;36(11):2130–6. doi: 10.1038/s41433-021-01815-4 (PMC9581986; doi:10.1038/s41433-021-01815-4)
Supplement: Supplementary file 2 — Changes in postoperative anterior chamber depth in eyes with and without posterior continuous curvilinear capsulorrhexis over time [file 41433_2021_1815_MOESM2_ESM.docx]

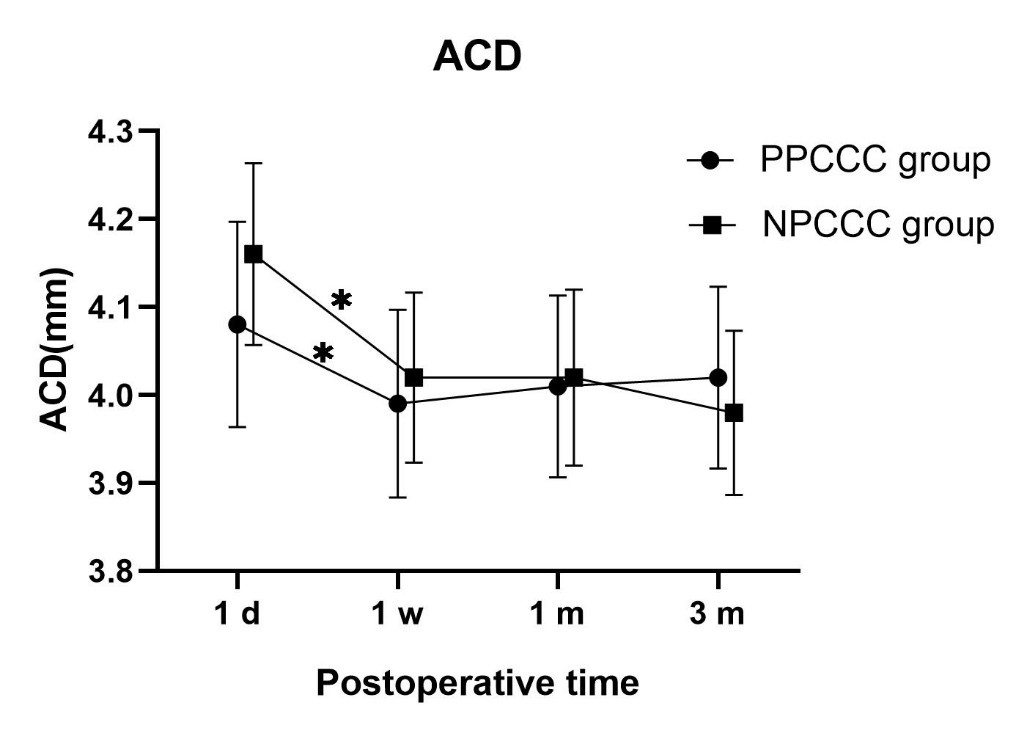


Figure S1. Changes in postoperative anterior chamber depth (ACD) in eyes with posterior continuous curvilinear capsulorrhexis(PPCCC) and without PPCCC(NPCCC) at 1 day, 1 week, 1 month, and 3 months postoperatively. Symbols and bars represent means and 95% confidence intervals, respectively. *The changes were statistically significant(P＜0.05).
